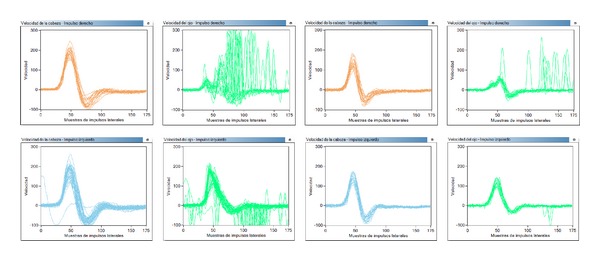# Vertigo as the First Sign of Chronic Myeloid Leukemia: A Case Report and Literature Review

**DOI:** 10.1155/2013/505636

**Published:** 2013-02-12

**Authors:** Rubén Martín-Hernández, Diego Hernando Macías-Rodríguez, Víctor Martín-Sánchez, Cristina Cordero-Civantos, Santiago Santa Cruz-Ruiz, Ángel Batuecas-Caletrio

**Affiliations:** Otoneurology Unit, Department of Otorhinolaryngology and Head and Neck Pathology, University Hospital of Salamanca, Paseo San Vicente 58-182, 37007 Salamanca, Spain

## Abstract

Acute vestibular deficit as the first sign of leukemia is extremely rare. The literature shows some cases of sudden hearing loss accompanied by instability and associated with hyperviscosity syndrome. We present the case of a patient who presents a harmonic vestibular deficit of the right ear. The complementary studies revealed an abnormally high level of leukocytes. A peripheral blood cytogenetic analysis is performed due to a high suspicion of leukemia, and the results show BCR/ABL fusion gene with a cut point in the M-BCR region, which confirms the diagnosis of chronic myeloid leukemia. In this case we detail the importance of taking hematological disorders into consideration in the differential diagnosis of patients with otoneurological symptoms, and we also review the etiopathogenic mechanisms, symptoms, diagnosis, and therapeutic options for chronic myeloid leukemia with sudden hearing loss and vertigo.

## 1. Introduction

Leukemia, both acute and chronic, does not usually appear with otoneurological symptoms as the first sign. The usual initial clinical symptoms are weakness, malaise, fever, unexplained weight loss, sleep hyperhidrosis caused by hypermetabolism, abdominal distension, dysphagia, and bone pain [1, 2].

On the contrary, most leukemia patients present, along the evolution of the disease, otological symptoms or signs (between 16% and 48%) [1, 3]. However, in global terms it is more common to observe otoneurological symptoms in patients with acute leukemia than in patients with chronic leukemia [4, 5]. 

Some cases of sudden sensorineural hearing loss as the first sign of chronic myeloid leukemia (CML) have been described, sometimes accompanied by vertiginous symptoms. In most cases, it is attributed to hyperviscosity syndrome [1, 2, 4].

Other causes of otoneurological symptoms in CML are middle and inner ear bleeding (cochlea, vestibule, and occupied perilymphatic space), tumor infiltrates, inflammation of the cochlea/vestibule, and damages of the middle ear, such as the destruction of the ossicles or perforation of the eardrum [1–3].

We present the case of a 24-year-old woman with no personal record of interest who was admitted as an emergency due to disequilibrium of two days of evolution. The exploration reveals a vestibular deficit harmonic syndrome in the right ear. The complementary tests show an abnormally high level of leukocytes, which pointed towards the possible origin of the vertiginous syndrome.

## 2. Case Presentation

The patient is a 24-year-old woman with no personal record of interest who reports disequilibrium and object spinning sensation of two days of evolution, accompanied by vagal symptoms; she reports tinnitus on the right ear but no hypoacusis or aural fullness.

The patient is conscious, oriented, eupneic, and with a slight skin paleness. The constants, neurological examination, ear examination, and electrocardiogram (EKG) were normal.

The vestibular examination reveals a grade II spontaneous nystagmus to the left without gaze fixation; Romberg's test, Unterberger-Fukuda test, and Bárány pointing test show a clear right deviation. The oculocephalic maneuver was positive for the right eye.

This exploration suggests an acute vestibular deficit in the right ear, and we decided to administer diazepam 5 mg i.v. in 30–45 minutes and to monitor the evolution while we wait for the results of an analysis that includes complete blood count, biochemistry, and basic coagulation tests. 

The treatment improves the symptoms of the patient, but the analysis shows Hb 7.3 g/dL, Leukocytes 572 × 10^9^/L, platelets 348 × 10^9^/L, prothrombine time (PT) 57; activated thromboplastine time (TTPA) 43.7, D-dimer 2.13.

The patient was referred to the department of hematology in order to proceed with the analysis. A peripheral blood smear reveals 5% of myeloid blasts, one erythroblast per 100 cells, and giant platelets. An exhaustive physical examination is performed, and it was found a splenomegaly that surpasses the midline and reaches the right side.

The patient is admitted in the department of hematology with high suspicion of CML and leukostasis syndrome. The cytogenetic study of the peripheral blood shows BCR/ABL fusion with a cut point in the M-BCR region, which confirms the diagnosis. The molecular biology test after a bone-marrow aspiration is RT-PCR positive for t(9;22) BCR/ABL with 7645cp.

It was decided to start treatment with hydration, allopurinol, and emergency leukapheresis (which is suspended when the level of leukocytes is <100^9^/L), and on the second day to start a cytoreductive therapy with hydroxycarbamide combined with rasburicase. When the leukocytes levels reached <50 × 10^9^/L, the hydroxycarbamide was suspended, and a treatment with nilotinib was started. It caused a toxicodermatitis that disappeared after the administration of antihistamines. The vertiginous symptoms progressively decreased until they disappeared during the first three days of hospitalization.

The abdominal ultrasound shows a spleen with normal morphology and structure, but with a large axis of 24 cm.

On the ninth day of hospitalization, the patient is discharged due to the improvement of the general and vertiginous symptoms and to the end of the diagnostic procedure, with leukocyte levels of 12 × 10^9^/L. There is still a grade II nystagmus with no fixation towards the left side.

**Figure 1:**
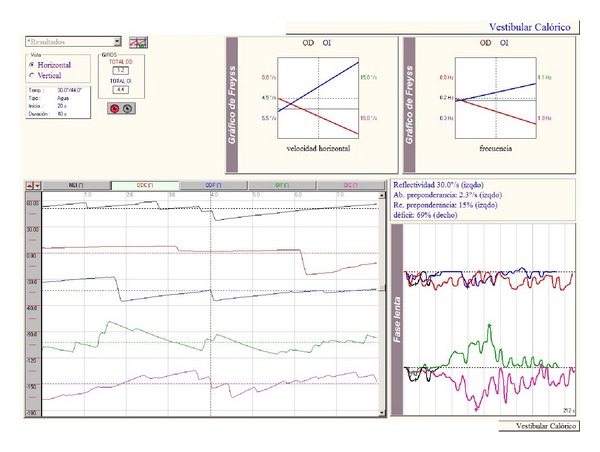


While monitoring the evolution in the outpatient visits to the ENT department, an audiometry revealed normal hearing. A videonystagmography (VNG) was performed two months after the acute process Figure 1, showing a right vestibular deficit of 69%, and the Nuclear Magnetic Resonance (NMR) was normal. A video head impulse test had been performed during the acute process and five months later, Figure 2, and the first one showed the presence of a complete destabilization of the oculomotor reflex, with the presence of overt and covert saccades in the context of a spontaneous nystagmus and a hearing gain below normal levels (0.20 for the pathological ear). Five months later, there is still a low gain for the right ear (0.38) with overt saccades but no covert saccades, as is usually the case in the context of vestibular compensation.

## 3. Discussion

Higher survival rates in CML patients thanks to the development of new therapies have led to a higher frequency of otoneurological symptoms in those patients, due to the fact that along their evolution they can present leukemic infiltration in the middle ear, cochlea, vestibule, and petrous apex. However, these symptoms are rare as a first sign [1, 6].

The pathogenesis of otoneurological alterations in chronic myeloid leukemia is complex. In any case, the associated hyperleukocytosis seems to play the most relevant role in this, as can be seen by the recovery of the patients after a leukapheresis [1, 2].

This syndrome is due to the formation of small leukocytic aggregates and thrombus that lead to infarction in different tissues.

The cases of incurable hearing losses even when the therapy was started precociously seem to be related to an occlusion of the labyrinthine artery due to leukostasis.

However, the mechanisms of leukostasis have not been completely clarified yet. In spite of the fact that it is attributed to an increase in the viscosity of microcirculation, some recent researches suggest that there may be an additional damage to the endothelium [4]. 

There are other possible causes of otoneurological symptoms in CML, such as, hemorrhage in the middle and inner ear, hemotympanum, tumor infiltrate, inflammation of the cochlea or the vestibulum, facial paralysis, destruction of the ossicles, perforated eardrum, and infection [1–3, 6].

We have to properly assess patients who apparently present clear repeated disorders, such as, otitis externa/media or facial paralysis, because leukemic patients are particularly prone to infections by *Staphylococcus aureus*, herpes zoster, aspergillosis, and mucormycosis. A chloroma (a localized mass of primitive myeloid cells of the granulocytic series that infiltrates extramedullary sites of a greenish hue) in the external auditory meatus can appear as an area with inflammatory and hemorrhagic changes [6, 7].

Obtaining diagnostic biopsies can be very difficult, especially in the middle ear, due to thrombocytopenia and consumption coagulopathy. However, CT scans and NMR imaging make it possible to distinguish between infection and tumor of the ear in some cases [6].

The reviewed literature shows no unanimity with regard to the most adequate therapy when CML starts with auditory or vestibular symptoms. In most reviewed cases, leukapheresis is performed every 12 hours until the levels of leukocytes decrease (as in our case); but in other cases, leukapheresis is not routinely applied [4], because it does not ensure an increase of the survival rates and/or hearing recovery, which may vary after the disorder is under control. If the hyperleukocytosis presents complications in the central nervous system or the respiratory system, leukapheresis must be performed, together with high doses of chemotherapy, because this combination has shown more effectiveness than conventional chemotherapy on its own.

In some cases, this therapy has been combined with initial intratympanic corticoids for hearing recovery, but it has shown no benefits or systemic worsening of the CML evolution [2].

The appearance of sudden sensorineural hearing loss makes it necessary to carry out a global assessment of the patient in order to rule out a systemic pathology before any therapy can be started, because the administration of corticoids in a hematologic disorder can trigger a leukemoid reaction [8].

When the complementary tests show cochlear leukemic infiltrate, the administration of intrathecal methotrexate is another effective treatment alternative [6, 9].

In some cases, the middle and inner ear can be difficult to reach by chemotherapy, and if a leukemic infiltrate appears in this area, the anatomical structure itself can act as a protection for tumor cells. In order to avoid this, sometimes radiotherapy is needed for a complete and lasting remission of the otoneurological symptoms [6].

The most characteristic features of our patient, compared with other published articles, is the presence of an harmonic vestibular deficit syndrome as the first sign of CML. We have not found any article that shows the presence of spontaneous nystagmus in the initial symptoms of CML, although many of them describe the feeling of instability and the presence of vagal symptoms. However, none of them present a harmonic deficit syndrome like the one of the patient we present in our study.

## 4. Conclusions

Hematologic disorders must be taken into account in the differential diagnosis of sudden hearing loss and vertigo, mainly due to the hyperviscosity syndrome that leads to alterations in the circulation of the vertebrobasilar area.

## Figures and Tables

**Figure 2:**